# Bridging therapy versus mechanical thrombectomy alone in patients with basilar artery occlusion and mild symptoms: a retrospective observational study

**DOI:** 10.1007/s00415-026-13883-1

**Published:** 2026-06-17

**Authors:** Simone Bellavia, Andrea M. Alexandre, Luca Scarcia, Giovanni Frisullo, Alessandro Pedicelli, Valerio Brunetti, Joseph D. Gabrieli, Valerio Da Ros, Antonio A. Caragliano, Orazio Buonomo, Vittorio Semeraro, Maria P. Ganimede, Giancarlo Salsano, Nicola Mavilio, Nicolas Chausson, Ghil Schwarz, Angelo Cascio Rizzo, Mariangela Piano, Stefano Caproni, Serena Abruzzese, Emilio Lozupone, Paolo Calabresi, Aldobrando Broccolini

**Affiliations:** 1 Neurology Unit, Fondazione Policlinico Universitario A. Gemelli IRCCS, Rome, Italy; 2 Interventional Neuroradiology Unit, Fondazione Policlinico Universitario A. Gemelli IRCCS, Rome, Italy; 3 Department of Neuroradiology, Henri Mondor Hospital, Creteil, France; 4 Neuroradiology Unit, Padua University Hospital, Padua, Italy; 5 Department of Biomedicine and Prevention, University Hospital of Rome “Tor Vergata”, Rome, Italy; 6 Neuroradiology Unit, A.O.U. Policlinico G. Martino, Messina, Italy; 7 Interventional Radiology Unit, P.O.C. SS Annunziata, Taranto, Italy; 8 Neuroradiology Unit, IRCCS Ospedale Policlinico San Martino, Genua, Italy; 9 Department of Neurology, Centre Hospitalier Sud Francilien, Corbeil-Essonnes, France; 10 Neurology Unit, ASST Grande Ospedale Metropolitano Niguarda, Milan, Italy; 11 Neuroradiology Unit, ASST Grande Ospedale Metropolitano Niguarda, Milan, Italy; 12 Neurology and Stroke Unit, Azienda Ospedaliera Santa Maria Di Terni, Terni, Italy; 13 Neuroradiology Unit, Hospital Vito Fazzi, Lecce, Italy; 14 Department of Neuroscience, Catholic University School of Medicine, Rome, Italy

**Keywords:** Ischemic stroke, Basilar artery occlusion,, Thrombolysis, Mechanical thrombectomy, Bridging therapy

## Abstract

**Background:**

The safety and efficacy of bridging therapy, which combines intravenous thrombolysis (IVT) with mechanical thrombectomy (MT), have not been fully explored in patients with basilar artery occlusion (BAO) and mild symptoms. This study aims to evaluate these factors in patients with BAO and an NIHSS score of less than 10.

**Methods:**

We conducted a multicenter retrospective observational study involving consecutive BAO patients with NIHSS < 10 from 10 European stroke centers between January 2017 and December 2024. We used inverse probability of treatment weighting (IPTW) to balance clinical variables. Primary safety outcomes included parenchymal hematoma (PH1/PH2) and in-hospital death. Secondary efficacy outcomes included successful recanalization after MT and the 90-day functional outcome.

**Results:**

A total of 134 patients were included. Bridging therapy did not increase the risk of PH (RR = 0.963; 95% CI 0.185–5.020; *p* = 0.965) or in-hospital death (RR = 0.620; 95% CI 0.174–1.980; *p* = 0.434). Additionally, IVT plus MT showed no significant improvement of the 90-day mRS score (ATE -0.187; 95% CI -1.0690.694; *p* = 0.675) or rates of 90-day mRS 0–2 (RR 1.192; 95% CI 0.483–3.011; *p* = 0.704) and 03 (RR 1.997; 95% CI 0.738–5.867; *p* = 0.186). No effect of IVT was observed on the rate of successful recanalization (RR 2.659; 95% CI 0.663–13.518; *p* = 0.190).

**Conclusions:**

Bridging therapy appears to be safe for BAO patients with mild symptoms, but it does not provide additional benefits for functional outcomes or efficient recanalization rates.

**Supplementary Information:**

The online version contains supplementary material available at 10.1007/s00415-026-13883-1.

## Background

Randomized controlled trials (RCTs), comparing the efficacy and safety of intravenous thrombolysis (IVT) followed by mechanical thrombectomy (MT)—commonly referred to as bridging therapy (BT)—versus MT alone, have focused on patients with anterior circulation stroke [1–6]. Evidence regarding the safety and efficacy of BT for basilar artery occlusion (BAO) remains limited, particularly in patients with mild symptoms. These patients were underrepresented in RCTs confronting MT with best medical management (BMM) alone for vertebrobasilar strokes, resulting in conflicting data concerning the overall benefit of MT in this subgroup [7–10]. Current guidelines advise against the use of MT as the first choice of treatment, yet recent works—based on real-world data—suggest a possible benefit of the endovascular approach also for these patients, at the cost of an increased bleeding risk [11]. Considering the therapeutic uncertainty, the possible additional benefit of MT has to be weighed against this bleeding risk, especially when combined with IVT. Nonetheless, the safety and efficacy of BT in stroke due to BAO are poorly explored. A recent registry study of patients with posterior circulation strokes found that BT was associated with better 90-day functional outcomes and lower mortality compared to MT alone, without safety concerns [12]. Nonetheless, the results came from a heterogeneous cohort, not limited to BAO cases. Similarly, a pooled meta-analysis of observational studies has shown that BT is associated with improved functional independence and reduced mortality in acute BAO, without increasing the risk of hemorrhagic complications [13]. Importantly, neither of these studies focused on patients with mild symptoms, nor are any data currently available regarding the safety and efficacy of BT over MT alone in this cohort. In consideration of this gap in the existing literature, the primary objective of this study was to evaluate the safety of BT in patients with BAO and a baseline NIHSS < 10, compared with MT alone. The secondary objective was to assess potential differences in efficacy outcome measures between these two treatment approaches.

## Methods

### Patients and treatment

This retrospective observational study involved 10 comprehensive stroke centers across Europe and was conducted within the framework of a non-profit study approved by the ethics committee of the Fondazione Policlinico Universitario A. Gemelli (protocol number 6410/20, ID 3004) and conducted in accordance with the Declaration of Helsinki. The local ethics committees approved the use of patients’ data, and the need for informed consent was waived due to the retrospective nature of the study. This analysis was conducted in adherence with the Strengthening the Reporting of Observational Studies in Epidemiology (STROBE) guidelines [14].

The prospective databases of participating centers were screened for patients with isolated BAO, a baseline NIHSS score < 10 and a pre-event mRS score < 3, who received MT between January 2017 and December 2024. Radiological assessment consisted of an initial non-contrast CT scan, followed by multiphase CTA to locate the site of occlusion. Basilar artery occlusion was defined as involving the basilar artery segment extending from the vertebral artery confluence to the basilar apex. The posterior circulation ASPECTS (pc-ASPECTS) was calculated on non-contrast CT. Demographics, cardiovascular risk factors and baseline clinical features were collected. Stroke etiology was classified according to the Trial of Org 10172 in Acute Stroke Treatment (TOAST) criteria [15]. Intravenous thrombolysis (IVT) was used in accordance with current guidelines and clinician decisions. Mechanical thrombectomy was conducted using a stent retriever, direct contact aspiration, or a combined technique. The initial MT technique and number of passes were recorded, with documentation of any subsequent techniques and corresponding pass numbers when the initial approach was unsuccessful. Successful recanalization at the end of MT was defined as a mTICI score ≥ 2b. Patients were divided into 2 groups based on prior administration of IVT before MT (IVT + MT group and MT-only group).

### Outcome measures

Intracranial hemorrhagic events were assessed on follow-up imaging within 72 h post-procedure. These were defined using the Heidelberg classification of Bleeding Events After Ischemic Stroke and Reperfusion Therapy [16] and divided into petechial (scattered-H1, or confluent-H2), parenchymal hematoma (PH) and subarachnoid hemorrhages (SAH). Parenchymal hematoma type 1 (PH1, occupying < 30% of the ischemic volume without mass effect) and parenchymal hematoma type 2 (PH2, occupying ≥ 30% of the infarcted tissue with mass effect) were merged into a single variable. This decision was based on the assumption that PH1 may also produce clinically relevant mass effect in the infarcted brainstem and thereby influence prognosis. Radiologic and angiographic data were reviewed locally by the neuroradiologists/neuro-interventionalists blinded to clinical information. Clinical outcome was measured with the mRS score acquired at 90 days by a trained physician.

The primary outcome measures were the occurrence of any post-procedural PH and in-hospital death. The secondary outcomes included 1) rates of successful recanalization, 2) the ordinal 90-day mRS score (mRS), and 3) the dichotomized 90-day mRS scores of 0–2 and 0–3.

### Statistical analysis

Differences in baseline characteristics and therapeutic procedures were assessed using the Mann–Whitney U test or Student’s t test for continuous variables and Pearson’s χ^2^ test for categorical variables. Bonferroni correction was applied to account for type I errors in case of multiple comparisons. Continuous variables are reported as median (interquartile range, IQR), and categorical variables as proportions. To adjust for potential confounders, an Inverse Probability of Treatment Weighting (IPTW) model was used to create a balanced dataset for the analysis. The model included covariates selected a priori for their clinical relevance or potential association with treatment allocation [sex, age, hypertension, pre-event mRS, admission NIHSS, time from last known well to groin, time from groin to recanalization, and baseline pc-ASPECTS], along with those imbalanced in univariate analysis (*p* < 0.05). Covariate balance was assessed by examining standardized mean differences (SMD), with values < 0.1 indicating acceptable balance. Treatment effects were assessed using a logistic regression model that also included the variables that remained unbalanced after IPTW, as well as those significantly different in the univariate analysis, if clinically relevant for the outcomes. This doubly robust approach, employing IPTW followed by logistic regression, ensured an accurate estimation of treatment effects, while adequately controlling confounding variables. Missing values were not imputed since no variable showed a missing record above 5%. Risk ratio (RR) or average treatment effect (ATE) and their 95% CIs were calculated for all models as appropriate, using the IVT + MT group as the reference. Multicollinearity among explanatory variables was assessed using the Variance Inflation Factor (VIF). For all analyses, significance was set at *p* < 0.05. All analyses were performed using R software version 4.1.3 with the *cobalt* and *causaldrf* packages [17, 18].

## Results

A total of 149 patients with BAO and admission NIHSS score < 10 were identified, of whom 134 were treated with endovascular therapy, with or without prior IVT. A flow diagram of patients’ identification and inclusion is summarized in Supplementary Fig. 1. Females were 61 (45.5%) and the median (IQR) age was 74 (60–83) years. The median (IQR) admission NIHSS score was 5 (3–8) and the median baseline pc-ASPECTS was 10 (8–10). 44 (32.8%) patients received IVT prior to MT. All relevant baseline information, including whether patients were directly referred to an EVT-capable center and reasons to withhold IVT treatment (whenever this data was available), is reported in Table 1. Successful recanalization was achieved in 90% of cases. Any intra-parenchymal hemorrhage occurred in 8.9% of patients, of whom 6.7% had a PH type 1 or 2. Overall, the median (IQR) 90-day mRS score was 1 (0–3) (Table 1).

**Table 1:** Demographic, clinical, radiological, procedural and outcome characteristics of the entire cohort

Age, median (IQR)	74 (60–83)
Female, # patients (%)	61/134 (45.5%)
*Clinical, radiological and procedural features*	
Pre-event mRS score, median (IQR)	0 (0)
Baseline NIHSS score, median (IQR)	5 (3–8)
Baseline pc-ASPECTS	10 (8–10)
Direct access to an EVT-capable center	102/134 (76.1%)
IVT plus MT, # patients (%)	44/134 (32.8%)
MT-only, # patients (%)	90/134 (67.2%)
Time from last known well to groin (minutes), median (IQR)	315 (219–458)
Groin to reperfusion time (minutes), median (IQR)	41 (27–70)
Reason to withhold IVT treatment	
Timing from last known well > 4.5 h	38/90 (42.2%)
Anticoagulant therapy	22/90 (24.4%)
Recent major trauma	4/90 (4.4%)
Recent surgery	4/90 (4.4%)
Recent ischemic stroke	4/90 (4.4%)
Recent major bleeding	1/90 (1.1%)
Active cancer	1/90 (1.1%)
Not specified	16/90 (17.7%)
First technique, # patients (%)	
ADAPT	104/134 (77.6%)
stent retriever	5/134 (3.7%)
combined	21/134 (15.7%)
not specified	4/134 (2.9%)
number of passes, median (IQR)	1 (1–1)
Second technique, # patients (%)	34/134 (25.4%)
ADAPT	5/34 (14.7%)
stent retriever	2/34 (5.9%)
combined	27/34 (79.4%)
number of passes, median (IQR)	1 (1–1)
mTICI 2b-3, # patients (%)	121/134 (90.3%)
*Outcomes*	
Hemorrhagic complications, # patients (%)	
Any parenchymal hemorrhage (TC/MRI 24–72 h)	12/134 (8.9%)
PH1/PH2 (CT/MRI 24–72 h)	9/134 (6.7%)
SAH (CT/MRI 24–72)	8/134 (5.9%)
In-hospital death	20/134 (14.9%)
90-day mRS score, median (IQR)	1 (0–3)
90-day mRS score 0–2, # patients (%)	95/134 (70.1%)
90-day mRS score 0–3, # patients (%)	103/134 (76.1%)

*pc-ASPECTS* posterior circulation ASPECTS, *IVT* intravenous thrombolysis, *MT* mechanical thrombectomy, *ADAPT* a direct aspiration first-pass technique, *PH* parenchymal hemorrhage, *SAH* subarachnoid hemorrhage, *IQR* interquartile range

In univariate analysis, the only significant differences were the admission NIHSS score, higher in the IVT + MT group [7 (5–8) vs 5 (3–7); *p* = 0.022] and a previous diagnosis of atrial fibrillation that was more frequent in the MT-only group [5/44 (11.4%) vs. 34/90 (37.8%); *p* = 0.002]. As expected, the IVT + MT group showed a significantly shorter time from last known well to groin [244 (181–308) minutes vs. 353 (266–592); *p* < 0.001], but also a shorter groin to reperfusion time [32 (20–60) minutes vs. 48 (30–71); *p* = 0.029] (Table 2). Inverse probability of treatment weighting was adjusted for these imbalanced variables, along with the other prespecified covariates; however, the admission NIHSS and the time from the last known well to groin remained slightly unbalanced (Supplementary Fig. 2). In a binary logistic regression model adjusted also for this residual unbalanced variable, the use of IVT before MT was not associated with a higher risk of PH occurrence [RR = 0.963; 95% CI 0.185–5.020; *p* = 0.965] or in-hospital death [RR = 0.620; 95% CI 0.174–1.980; *p* = 0.434] (Table 3). Similarly, IVT plus MT showed no effect on the ordinal 90-day mRS score [ATE -0.187; CI -1–069- 0.694; *p* = 0.675] nor on the dichotomized 90-day mRS 0–2 [RR 1.192; 95% CI 0.483–3.011; *p* = 0.704] and 0–3 [RR 1.997; 95% CI 0.738–5.867; *p* = 0.186] (Table 3). There was also no effect of prior IVT on the rate of  successful recanalization [RR 2.659; CI 0.663–13.518; p = 0.190] (Table 3). Complete results of the regression models are presented in Supplementary Table 1 (Supplementary Table 1).

**Table 2:** Univariate analysis confronting the IVT-plus-MT with the MT-only groups

Variables	IVT + MT (44)	MT-only (90)	p value
Age, median (IQR)	71 (59–79)	76 (62–83)	0.138
Female, # patients (%)	21/44 (47.7%)	40/90 (44.4%)	0.634
Pre-event mRS score, median (IQR)	0 (0)	0 (0)	0.339
Admission NIHSS score, median (IQR)	7 (5–8)	5 (3–7)	**0.022**
Atrial Fibrillation, # patients (%)	5/44 (11.4%)	34/90 (37.8%)	**0.002**
Hypertension, # patients (%)	27/44 (61.4%)	69/90 (76.7%)	0.095
Diabetes, # patients (%)	9/44 (20.5%)	17/90 (18.9%)	0.781
Coronary artery disease, # patients (%)	8/44 (18.2%)	19/90 (21.1%)	0.511
*TOAST classification, # patients (%)*			
large artery atherosclerosis	19/44 (43.2%)	25/44 (27.7%)	0.060
cardioembolic	15/44 (34.1%)	46/90 (51.1%)	**0.044**
other determined etiologies	2/44 (4.5%)	2/44 (2.2%)	0.443
cryptogenic	8/44 (18.2%)	17/44 (18.9%)	0.969
pc-ASPECTS baseline, median (IQR)	10 (9–10)	10 (8–10)	0.186
Direct access to an EVT-capable center	34/44 (77.3%)	68/90 (75.6%)	0.520
Time from last known well to groin (minutes), median (IQR)	244 (181–308)	353 (266–592)	** < 0.001**
Groin to reperfusion time (minutes), median (IQR)	32 (20–60)	48 (30–71)	**0.029**
*First technique, # patients (%)*			
ADAPT	36/44 (81.8%)	68/90 (75.6%)	0.451
stent retriever	1/44 (2.3%)	4/90 (4.4%)	0.548
combined	4/44 (9.1%)	17/90 (18.8%)	0.156
not specified	3/44 (6.8%)	1/90 (1.1%)	
number of passes, median (IQR)	1 (1–1)	1 (1–1)	0.340
*Second technique, # patients (%)*			
ADAPT	2/44 (4.5%)	3/90 (3.3%)	0.709
stent retriever	-	2/90 (2.2%)	0.325
combined	9/44 (20.5%)	18/90 (20.0%)	0.849
number of passes, median (IQR)	1 (1–2)	1 (1–2)	0.731
mTICI (2b-3), # patients (%)	41/44 (93.2%)	81/90 (90.0%)	0.569
Any IPH (TC/MRI 24–72 h), # patients (%)	2/44 (4.5%)	10/90 (11.1%)	0.083
PH1/PH2 occurrence, # patients (%)	2/44 (4.5%)	7/90 (7.8%)	0.211
SAH occurrence, # patients (%)	2/44 (4.5%)	6/90 (6.7%)	0.637
In-hospital death, # patients (%)	5/44 (11.4%)	15/90 (16.7%)	0.543
90-day mRS score, median (IQR)	1 (0–3)	1 (0–4)	0.966
90-day mRS score 0–3, # patients (%)	37/44 (84.1%)	66/90 (73.3%)	0.166
90-day mRS score 0–2, # patients (%)	32/44 (72.7%)	63/90 (70.0%)	0.706

*pc-ASPECTS* posterior circulation ASPECTS, *IVT* intravenous thrombolysis, *EVT* endovascular  therapy, *MT* mechanical thrombectomy, *ADAPT* a direct aspiration first-pass technique, *PH* parenchymal hemorrhage, *SAH* subarachnoid hemorrhage, *IQR* interquartile range

**Table 3:** Doubly Robust Regression Analysis

	ATE	RR	95%CI	p value
*Safety outcomes*				
PH1/PH2	–	0.963	0.185–5.020	0.965
In-hospital death	–	0.620	0.174–1.980	0.434
*Efficacy outcomes*				
mTICI 2b-3 score	–	2.659	0.663–13.518	0.190
90-day mRS score	−0.187		−1.069–(0.694)	0.675
90-day mRS 0–2	–	1.192	0.483–3.011	0.704
90-day mRS 0–3	–	1.997	0.738–5.867	0.186

The regression model was adjusted for Inversion Probability of Treatment Weighting unbalanced variables (admission NIHSS and time from last known well to groin) and significantly different variables in univariate analysis (atrial fibrillation)

*PH1/PH2* parenchymal hematoma type 1/2; *RR* risk ratio, *ATE* average treatment effect

## Discussion

Our multicenter retrospective study suggests that prior administration of IVT is not associated with an increased risk of parenchymal hematoma or in-hospital death in patients with BAO and baseline NIHSS < 10, compared to MT alone. In addition, we observed no effect of BT on the rate of successful recanalization or 90-day functional outcomes.

Previous studies have investigated BT in posterior circulation stroke. A patient-data meta-analysis of four randomized trials on MT in BAO found no effect of BT on the 90-day mRS score, with a similar rate of sICH compared to MT alone [19]. Conversely, another meta-analysis reported that BT was associated with improved functional independence and reduced mortality, without increasing sICH risk [20]. However, neither of these studies reported a separate analysis for BAO patients with mild symptoms. Conversely, a recent meta-analysis comparing BT with MT alone included a focused analysis of patients with BAO and admission NIHSS scores between 5 and 15. The results were consistent with those of the overall cohort, showing that IVT plus MT was associated with higher odds of independent ambulation without an increased risk of sICH [13]. Overall, our data align with existing evidence supporting the safety of BT in patients with BAO and extend these findings to those with mild symptoms. In contrast, we did not observe an effect of BT on 90-day clinical outcomes. One possible explanation is that, in patients with mild symptoms, recanalization with MT alone may be sufficient to achieve near-maximal recovery, thereby negating any additional benefit of IVT. This interpretation is supported by data from clinical trials in anterior circulation stroke, in which BT was not superior to MT alone with respect to 90-day functional outcomes, even among patients with mild symptoms [21]. However, it is also important to note that IVT in our study was administered exclusively with alteplase, whereas emerging evidence suggests that tenecteplase in combination with MT may further enhance recanalization rates in patients with BAO [22]. In this context, the recent Randomized Trial of Thrombectomy with versus without Recombinant Human Tenecteplase Tissue Plasminogen Activator in Stroke (BRIDGE TNK), which compared tenecteplase-based BT with MT alone, reported higher rates of functional independence in the BT group. Nevertheless, given the limited number of vertebrobasilar patients enrolled, the generalizability of these findings to BAO patients, and particularly those presenting with mild symptoms, remains limited [23]. In this regard, our results apply to alteplase-based BT and should not be extrapolated to tenecteplase-based strategies.

Our work has several limitations, primarily due to its retrospective design. Although clinical and procedural records were carefully reviewed, the results could be influenced by data quality constraints inherent to non-randomized studies. Because treatment allocation was not randomized and despite IPTW, a potential selection bias cannot be excluded, whereby patients treated with MT alone may have been perceived as having a higher bleeding risk based on unmeasured clinical factors, while those receiving IVT prior to MT may have been considered at lower hemorrhagic risk; such imbalance may have contributed to the neutral findings observed in the comparison between IVT plus MT and MT alone in this population of patients. In addition, imaging data were assessed locally and not evaluated by a central core laboratory. Another limitation of our study is that it does not expand the available evidence comparing BMM alone with MT in BAO patients with mild symptoms, which is currently the critical topic of investigation concerning these patients. This may affect the clinical relevance of our findings although exploring the safety and efficacy of bridging therapy in BAO may help inform clinical decisions in light of this therapeutic uncertainty. Furthermore, patients who achieved recanalization prior to endovascular treatment (EVT) may have been excluded from the analysis as they were considered among those treated with BMM-only. This may have introduced an additional selection bias to our work. Finally, although our study has, to our knowledge, the largest cohort of patients with BAO and mild symptoms in which bridging therapy was compared with MT alone, the limited absolute sample size may have precluded our ability to detect meaningful differences between patients treated with or without prior IVT.

## Conclusion

Patients with basilar artery occlusion and mild symptoms represent a rare subgroup for whom optimal therapeutic management remains debated. Bridging therapy appears safe, with no increased risk of parenchymal hematoma compared with MT alone; however, no additional benefit in functional or recanalization outcomes was observed. Our findings should be interpreted with caution and warrant confirmation in large, prospective randomized trials specifically targeting BAO patients with mild symptoms.

## Supplementary Information

Below is the link to the electronic supplementary material.

Supplementary file1 (DOCX 207 KB)

## Data Availability

Data are available upon reasonable request. The anonymized data that support the results of this study are available on request from the corresponding author.
