# Supplementary material for: Bridging therapy versus mechanical thrombectomy alone in patients with basilar artery occlusion and mild symptoms: a retrospective observational study
Source: J Neurol. 2026 Jun 17;273(7):403. doi: 10.1007/s00415-026-13883-1 (PMC13275590; doi:10.1007/s00415-026-13883-1)
Supplement: Supplementary file 1 — Supplementary file1 (DOCX 207 KB) [file 415_2026_13883_MOESM1_ESM.docx]

**BRIDGING THERAPY VERSUS MECHANICAL THROMBECTOMY ALONE IN PATIENTS WITH BASILAR ARTERY OCCLUSION AND MILD SYMPTOMS: A RETROSPECTIVE OBSERVATIONAL STUDY**

**Supplementary Material**

**Supplemental Figure 1. Flow diagram of patients selection**

**
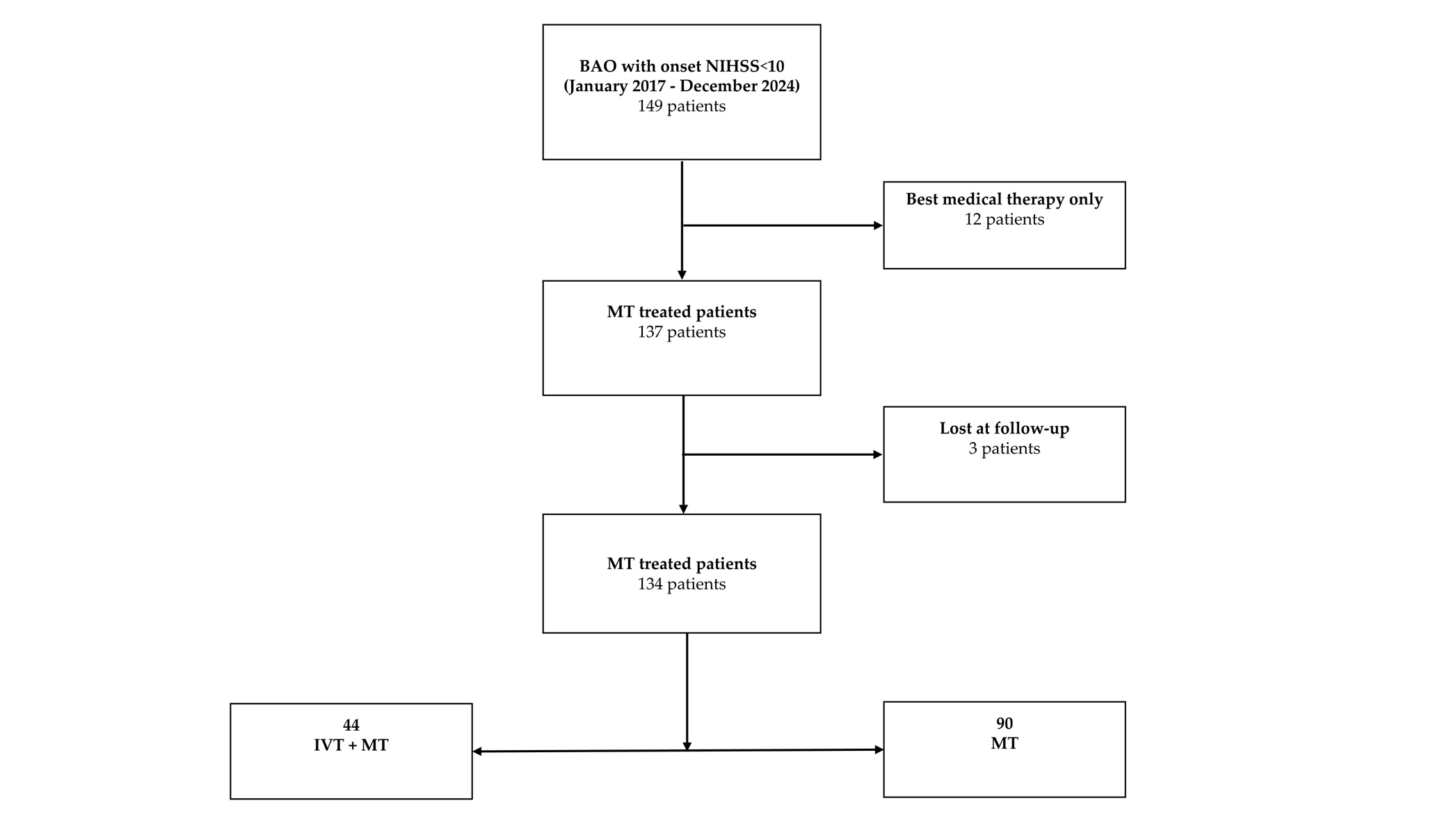
**

BAO, basilar artery occlusion; MT, mechanical thrombectomy; IVT, intravenous thrombolysis.

**Supplementary Figure 2. Plot of standardized mean difference of covariates before and after**

**propensity score matching**

**
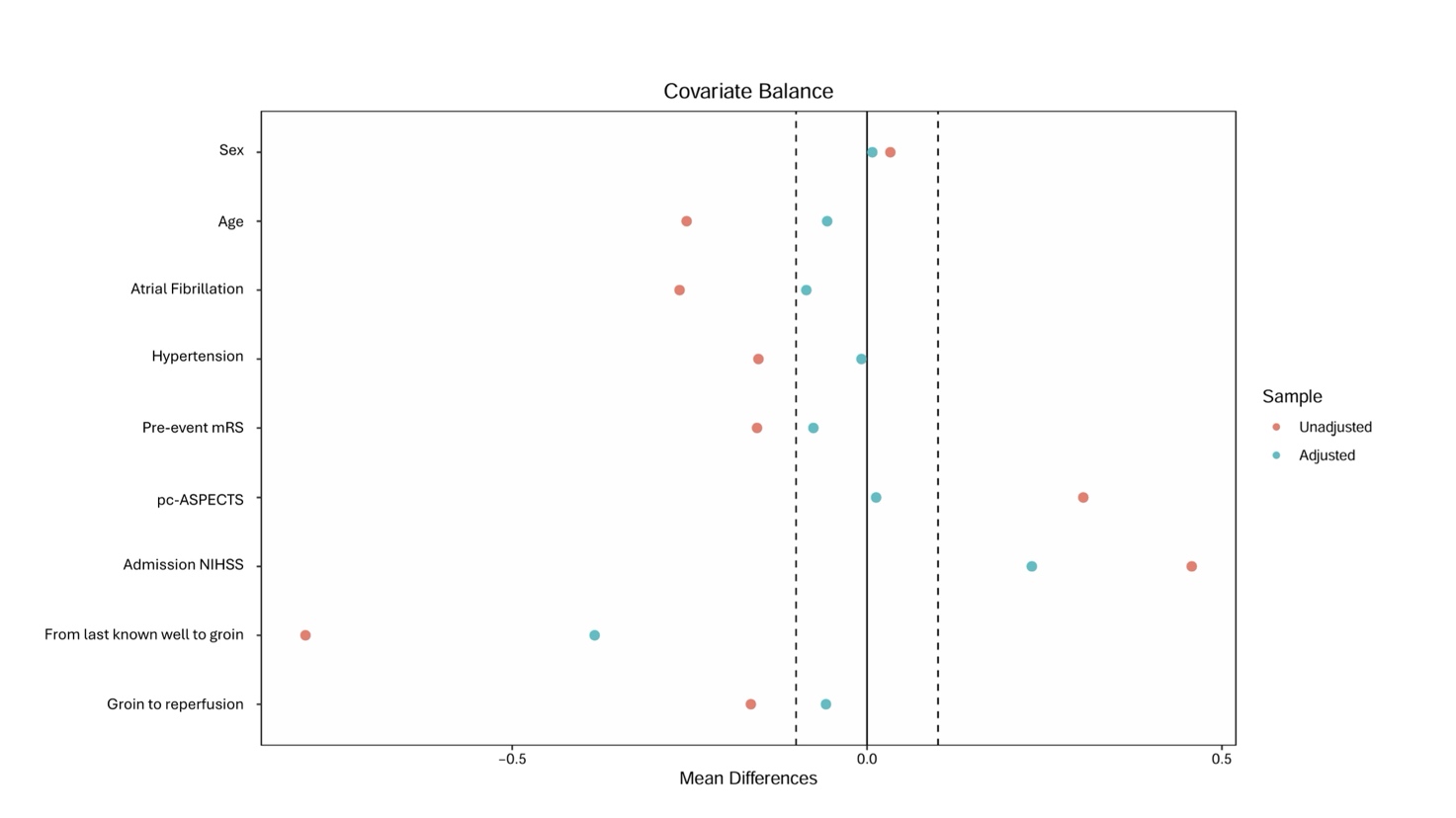
**

pc-ASPECTS, posterior circulation ASPECTS.

| **PH1/PH2** | | | | |
| --- | --- | --- | --- | --- |
| **Variable** | **ATE** | **RR** | **95%CI** | **p-value** |
| IVT+MT |  | 0.963 | 0.185-5.020 | 0.965 |
| Atrial Fibrillation |  | 3.967 | 1.307-12.033 | 0.015 |
| Admission NIHSS score |  | 1.135 | 0.784-1.641 | 0.498 |
| From last known well to groin time |  | 1.001 | 0.998-1.003 | 0.412 |
|  | | | | |
| **In-hospital death** | | | | |
| IVT+MT |  | 0.620 | 0.174-1.980 | 0.434 |
| Atrial Fibrillation |  | 1.034 | 0.325-3.014 | 0.952 |
| Admission NIHSS score |  | 1.214 | 0.997-1.505 | 0.062 |
| From last known well to groin time |  | 1.001 | 0.999-1.002 | 0.355 |
|  | | | | |
| **90-day mRS score** | | | | |
| IVT+MT | -0.187 |  | -1.069 – (0.694) | 0.675 |
| Atrial Fibrillation | 0.336 |  | -0.565 – (1.238) | 0.462 |
| Admission NIHSS score | 0.143 |  | -0.015 – (0.302) | 0.076 |
| From last known well to groin time | -0.008 |  | -0.002-(0.002) | 0.926 |
|  | | | | |
| **90-day mRS score 0-2** | | | | |
| IVT+MT |  | 1.192 | 0.483-3.011 | 0.704 |
| Atrial Fibrillation |  | 0.621 | 0.265-1.465 | 0.272 |
| Admission NIHSS score |  | 0.871 | 0.742-1.014 | 0.081 |
| From last known well to groin time |  | 1.000 | 0.998-1.002 | 0.904 |
|  | | | | |
| **90-day mRS score 0-3** | | | | |
| IVT+MT |  | 1.997 | 0.738-5.867 | 0.186 |
| Atrial Fibrillation |  | 0.920 | 0.374-2.352 | 0.858 |
| Admission NIHSS score |  | 0.893 | 0.753-1.050 | 0.177 |
| From last known well to groin time |  | 0.999 | 0.998-1.001 | 0.640 |
|  | | | | |
| **mTICI 2b-3** | | | | |
| IVT+MT |  | 2.659 | 0.663-13.518 | 0.190 |
| Atrial Fibrillation |  | 2.701 | 0.617-19.155 | 0.234 |
| Admission NIHSS score |  | 0.757 | 0.561-0.978 | 0.046 |
| From last known well to groin time |  | 1.001 | 0.998-1.004 | 0.509 |
|  | | | | |

**Supplementary Table 1:** **Complete logistic regression models for safety and efficacy outcomes in the whole cohort.**

PH, parenchymal hematoma; IVT, intravenous thrombolysis; MT, mechanical thrombectomy.
